# Silver and Copper Complexes with Ibuprofen and Caffeine—Preparation and Evaluation of Their Selected Biological Effects

**DOI:** 10.3390/molecules29020506

**Published:** 2024-01-19

**Authors:** Anna Borówka, Anna Sierosławska, Andrea Baier, Anna Rymuszka, Elżbieta Olszewska

**Affiliations:** 1 Department of Animal Physiology and Toxicology, The John Paul II Catholic University of Lublin, Konstantynów Str. 1i, 20-708 Lublin, Polandanrym@kul.pl (A.R.); 2 Department of General and Coordination Chemistry and Crystallography, Faculty of Chemistry, Maria Curie-Sklodowska University in Lublin, Maria Curie-Sklodowska Sq. 3, 20-031 Lublin, Poland

**Keywords:** metal complexes, copper, silver, ibuprofen, caffeine, cytotoxicity, antimicrobial effects

## Abstract

Several organometallic complexes based on more than twenty different metals have already been approved for medical applications. The aim of the presented research was to obtain complexes of silver and copper with the non-steroidal anti-inflammatory drugs ibuprofen and xanthine alkaloid caffeine and evaluate selected aspects of their bioactivity and biosafety in terms of their future possible applications. The obtained complexes were characterized by Fourier-transform infrared spectroscopy, thermogravimetry, UV-VIS spectroscopy, conductometry, elemental analysis, and bioassays. Cytotoxicity for normal human cells of the CCD-Co18 cell line was evaluated by determining the IC_50_ value, with metabolic and morphology assessments. It was observed that complexes containing ibuprofen and caffeine exhibited lower toxicity than those with ibuprofen only. Complexes with copper showed lower toxicity towards healthy human fibroblasts compared to silver-based compounds, with an IC_50_ above 140 μg mL^−1^. However, in the silver complexes, the presence of caffeine increased the potency of COX-2 inhibition. Antimicrobial effects against different Gram-positive and Gram-negative bacterial strains were evaluated by MIC determination with values less than 20 μg mL^−1^.

## 1. Introduction

The non-steroidal anti-inflammatory drugs (NSAIDs) are a class of medicaments of antipyretic, anti-inflammatory, and analgesic activity. Ibuprofen (IBU), (RS)-2-(4-(2-methylpropyl)phenyl)propanoic acid, acting as a non-selective cyclooxygenase (COX) inhibitor, is among the most commonly used NSAIDs. Since drug absorption depends on the dissolution rate and IBU, a carboxylic acid, has low solubility in water, attempts have been made to obtain a drug form with better pharmacokinetic properties [1,2]. Among others, attempts to combine IBU with caffeine (CAF) have been made, which resulted in significantly increased IBU solubility in water [2]. Because both ingredients have specific biological activity, their combination may produce a number of beneficial effects. The disadvantage of IBU is that long-term use and the resulting COX-1 inhibition may cause negative changes in the body, especially in the digestive system. Therefore, opportunities to reduce these adverse effects are very important.

CAF, a methylxanthine alkaloid, belongs to the group of well-known bioactive substances that act by blocking adenosine receptors. CAF is able to form complexes with different acidic drugs and modify their pharmacokinetic properties [3]. Depending on the drug, it can act as a metabolic activator or inhibitor by interactions with hepatic cytochrome P-450 and P-448 enzymes, leading to the modification of the rate of drug elimination. It was also demonstrated to increase drug blood levels or affect the excretion of some drugs [3]. CAF has been approved in the treatment of respiratory problems in premature infants; additionally, it is used in treating headaches caused by various factors, and there have been attempts to use it to treat depression and neurocognitive decline, including that observed in Alzheimer’s and Parkinson’s disease [4]. Efforts are also being made to use CAF as a factor supporting anticancer therapy. There are reports proving that CAF has anticancer properties through blocking the post-replication repair of induced DNA damage in cancer cells; it intensifies the effects of the cis-platin, induces apoptosis, and suppresses proliferation in several types of cancer or permanently inactivates breast stromal myofibroblasts and inhibits their paracrine procarcinogenic activity [5,6,7,8]. Moreover, the combination of CAF with, e.g., acetylsalicylic acid, was able to intensify its analgesic effect [9].

There are a number of side effects associated with the clinical use of NSAIDs [10]. Many attempts have been made to reduce them, and one successful strategy has been the application of d-block metal complexes with NSAIDs. The concept of using metal complexes for medical functions is constantly being developed and implemented. It has been observed that some d-block metal ions, such as Cu(II), Zn(II), and Ag(I), can themselves act as anti-inflammatory agents [11]. Interactions between different metals and biomolecules provide diverse options to influence their properties; speciation; reactivity; and, finally, their biological effects [12]. Metal ions coordinated with NSAIDs provide advantages over the drugs themselves. The metal complexes of NSAIDs display a range of biological activities quite often inaccessible to the original NSAID ligands. This is because the NSAID–metal complexes exhibit molecular properties different from those of the parent drugs [13]. The interactions between metal ions and NSAIDs may lead to new desirable features, e.g., the Zn(II) complex of aspirin has a better therapeutic index than aspirin itself, exhibiting high stability, low cytotoxicity, and high biocompatibility [14]. The formation of appropriate complexes of metals with other substances may enhance their desired biological effect, improve their pharmacodynamic properties, enhance their bioavailability, and reduce the potential undesired toxic effects of metal ions [15]. 

For many years, studies of copper(II) complexes with various drugs have been the subject of bioinorganic chemistry research, because Cu is a component of many key proteins [16] and due to the high activity of copper against bacteria, viruses, yeasts, and fungi [17]. Particularly, copper(II) complexes with IBU have been widely described in the literature. Trinchero et al. [18], as well as Shahabadi and Shiri [19], reported their spectroscopic behavior, while Kowcun et al. [20] studied the influence of various sources of copper ions on the structure of the complex molecules. Kaur et al. [21] estimated their stability constants, and thermal investigations were conducted by Tita et al. [22]. In vitro anti-inflammatory studies and the DNA interaction and Density Functional Theory modeling of IBU complexes with various metals were conducted by Abbas et al. [23]. Malis et al. [24] described in vitro and in silico biological profiles of copper(II) complexes with NSAIDs. 

Silver is also known as an antibacterial and antifungal agent. Djokić [25] determined that only silver in ionic or complexed form is antimicrobially active, while elemental silver is not active. Conversely, Amin et al. [26] reported the antimicrobial properties of silver nanoparticles functionalized with 4-(phenylsulfonamido)benzoic acid (PSBA). Some silver(I) complexes with IBU have already been synthesized and tested. On the basis of Density Functional Theory calculations, Pereira e Silva et al. [27] proposed a dimeric silver bridging structure for the Ag-IBU complex. On the contrary, Kafarska and Wolf [28] obtained an Ag(I) complex with IBU as a monodentate ligand. Its thermal stability was very close to that of the free IBU. Naglah et al. [29] reported that complexation between the metal ions Ag(I) and Cu(II) and IBU yielded a molar ratio of 1:1 and 1:2 (metal:ibuprofen), respectively. They exhibited a significant effect against some bacteria and fungi. The bidentate interaction through the deprotonated carboxylate group of IBU with Ag(I) and Cu(II) was confirmed by Nunez et al. [30].

Although the use of metal complexes in the treatment of various diseases is increasing, they require further research in order to optimize the preparation of their appropriate forms. This paper presents the synthesis of binary and ternary complexes of copper(II) and silver(I) ions with IBU and CAF. A ternary complex compound of Ag(I) ions with CAF and IBU was obtained and studied for the first time. The physicochemical properties of all synthesized complexes were analyzed. Additionally, the obtained compounds were tested for cytotoxicity and pro-inflammatory side effects. The anti-inflammatory effect, understood as the ability of the complexes to inhibit the activity of cyclooxygenases, and the potential antibacterial effect were also assessed. 

## 2. Results and Discussion

### 2.1. Synthesis and Characterization

The complexes were prepared by the reaction of the appropriate salt (copper sulphate (Cu) or silver nitrate (Ag)) with ibuprofen sodium salt (IBU-Na) or IBU-Na and CAF in a water–methanol solution (see Materials and Methods). The synthesized complexes with silver were white in color, while those with copper were grayish green. The solids obtained were practically insoluble in water and methanol but quite well soluble in dimethyl sulphoxide (DMSO).

**Figure 1:**
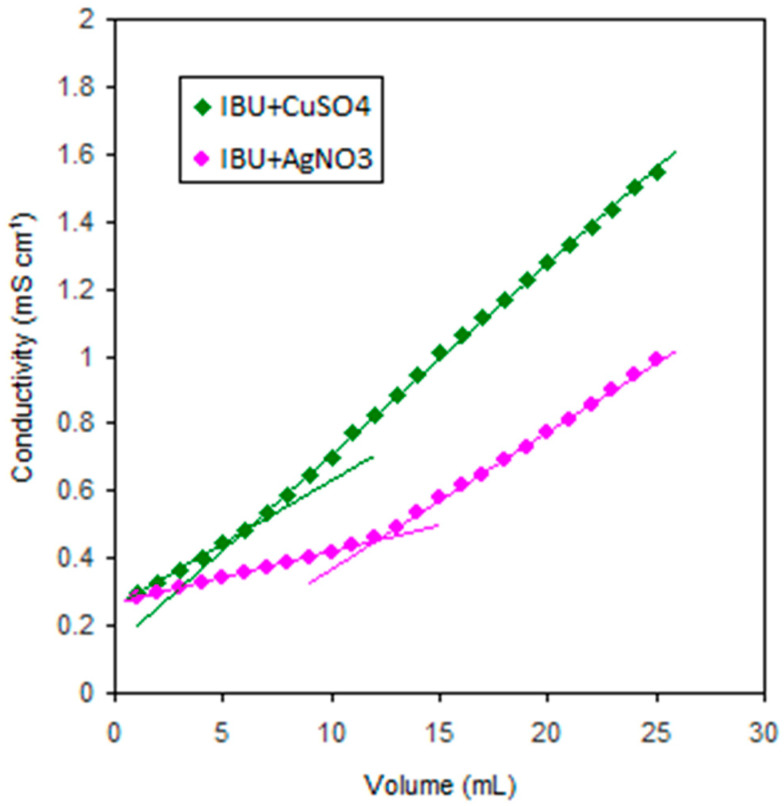
Conductometric titration curves for aqueous solutions of IBU-Na and CuSO_4_ or AgNO_3_.

Conductometric titration was used to determine the IBU:metal ratio in the molecules of the obtained complex compounds. Figure 1 shows the titration curves for 25 mL of 0.005 M aqueous IBU-Na solution against 0.01 M aqueous solutions of silver and copper salts.

From the inflection points of the straight lines on the graph, it can be concluded that complexes with silver formed in a ratio of 1:1 (Ag:IBU) and those with copper in a ratio of 1:2 (Cu:IBU).

**Figure 2:**
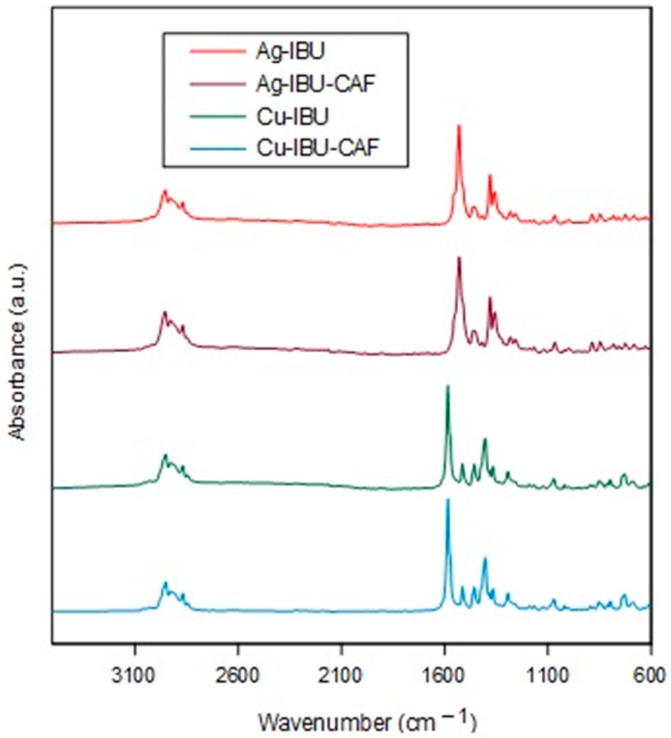
FTIR spectra of complexes Ag-IBU, Ag-IBU-CAF, Cu-IBU, and Cu-IBU-CAF in the range of 3500–600 cm^−1^. Several spectra were shifted along the Y-axis.

In order to study the mode of metal:ligand connection, Fourier-transform infrared spectroscopy was used. The spectra obtained by the FTIR method for the investigated complexes are shown in Figure 2.

The FTIR spectra of all compounds were evaluated to detect the coordination sites implicated in complexation. The bands of the aromatic and aliphatic (C-H) stretching modes appeared in the range 3000–2800 cm^−1^. The absence of some bands of absorption in the region 3500–3000 cm^−1^, characteristic for ν_O-H_ vibrations, confirmed the fact that the obtained complex did not contain water molecules [22]. In the spectral region of 1650–1300 cm^−1^, the asymmetric (ν_asym_) and symmetric (ν_sym_) stretching vibrations of the carboxylated ion were present. The IR spectrum of sodium ibuprofenate (parent salt) exhibited in this region two strong bands at 1581 and 1400 cm^−1^ (not shown), assigned to the asymmetric and symmetric stretching modes, respectively [31]. According to the Nakammoto criteria [32], the value of Δν = ν_asym_ − ν_sym_ and the direction of the band shifts in comparison to the corresponding values in the parent sodium salt characterized the nature of the metal–carboxylate bond. If the coordination is monodentate, the ν_asym_ and ν_sym_ frequencies are shifted to higher and lower values, respectively, and the Δν for Na-IBU is lower than the Δν for complex compounds. In the case of Δν_Na-IBU_ > Δν_complex_, the COO^−^ group is a bidentate chelating donor, and for Δν_Na-IBU_ ≈ Δν_complex_ it acts as a bidentate bridging donor. The wavenumbers for the ν_asym_ and ν_sym_ stretching modes of carboxylic groups in ibuprofen sodium salt and the obtained complexes are collected in Table 1.

Based on the above facts and the comparison of the frequencies given in Table 1, it can be concluded that the IBU in the synthesized complexes with copper is a bidentate bridging donor, while in silver complexes, the carboxylic group is a bidentate chelating donor. Similar results for Cu-IBU complexes were obtained by Naglah et al. [29] and Tita et al. [22], and for Ag-IBU compounds by Pereira e Silva et al. [27]. 

**Figure 3:**
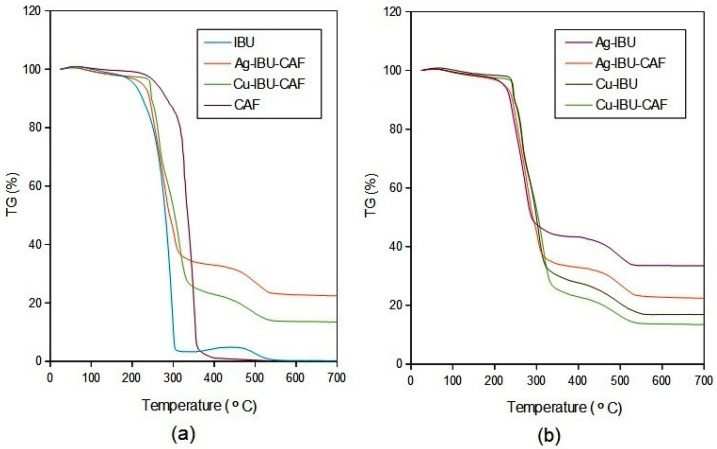
Thermogravimetric curves for: (**a**) IBU, CAF, and ternary complexes of Ag(I) and Cu(II); (**b**) binary and ternary complexes with IBU and CAF.

The composition of the obtained complex compounds was also confirmed using the method of thermal analysis. Thermogravimetric data (Figure 3) show that the decomposition processes of all complexes began above 200 °C, at temperatures between those of pure IBU and CAF. 

The decomposition of the silver complexes began at a slightly lower temperature than the copper complexes. Their pyrolysis proceeded in a similar manner in three stages. It is difficult to describe the nature of the intermediate compounds due to simultaneous and competitive reactions taking place. The final product here was a silver-white solid. The residue was 33.5% and 21.9% for the binary and ternary silver complexes, respectively. It corresponded to the content of pure silver, assuming a 1:1 combination of Ag-IBU and Ag-IBU-CAF in a 1:1:1 ratio, where the calculated amounts were 34.5% and 21.3%, respectively. The same composition of Ag-IBU was reported by Kafarska and Wolf [28]. 

The pyrolysis of the copper complexes proceeded in four steps, and the final product was a black powder, suggesting the formation of copper(II) oxide, which is stable even above 700 °C. The conversion of the resulting residue masses (16.9% and 12.9%) to pure copper content gave a result corresponding to the combination of the constituents into a Cu-IBU compound in a ratio of 1:2 and Cu-IBU-CAF in a ratio of 1:2:1. A similar Cu-IBU compound was obtained by Trinchero et al. [18]. In addition, the lower thermal stability of the complexes with silver compared to those with copper confirmed the ability of silver ions to act as an oxidation promoter.

**Figure 4:**
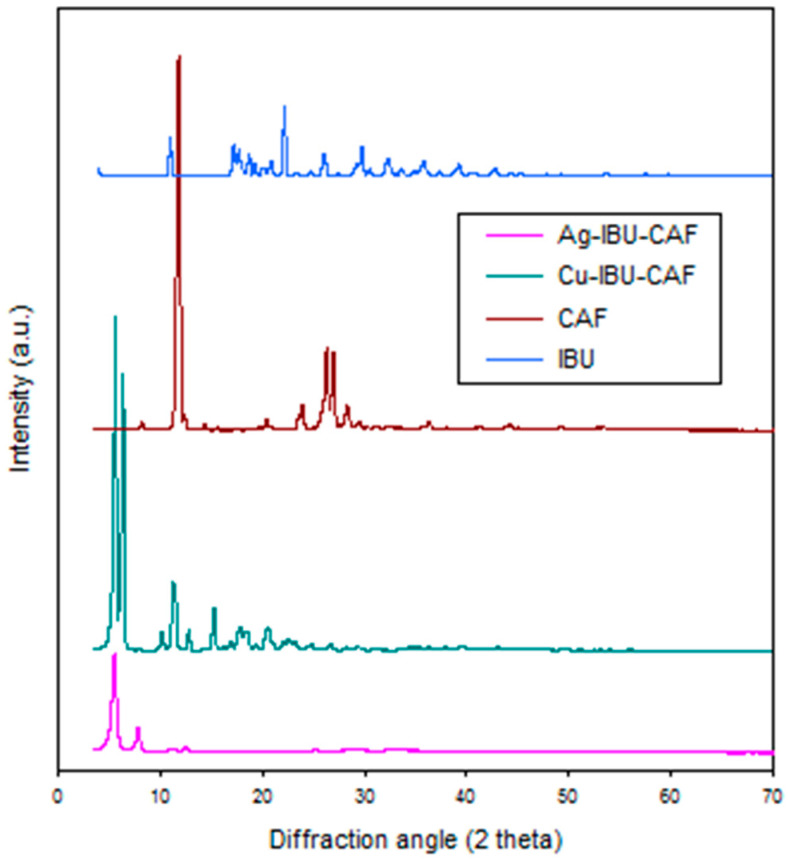
X-ray diffraction patterns of IBU, CAF, Cu-IBU-CAF, and Ag-IBU-CAF.

The complexes were precipitated during the synthesis as fine powders, and our attempts to isolate their monocrystals suitable for single-crystal X-ray diffractometry were unsuccessful. However, the recrystallization of these materials with DMSO could lead to other structures. Therefore, the formation of new ternary compounds with crystallographic structures different from the starting substances was confirmed using powder X-ray diffraction (Figure 4).

**Scheme 1:**
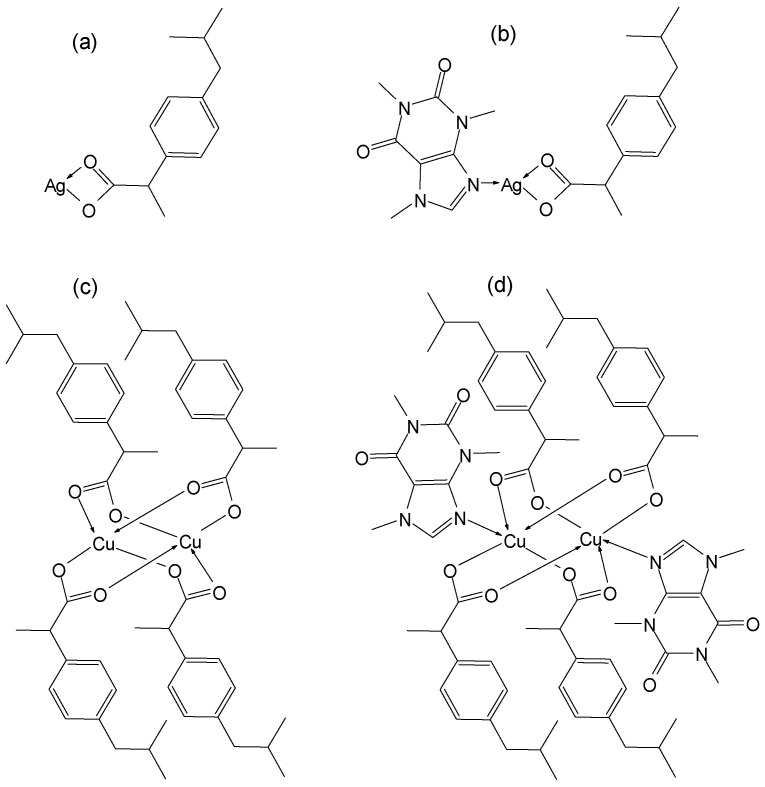
Structures of: (**a**) Ag-IBU; (**b**) Ag-IBU-CAF; (**c**) Cu-IBU; and (**d**) Cu-IBU-CAF.

Taking into account all the above data, it was possible to determine the molecular formulae of the complexes obtained. The binary and ternary compounds with Cu(II) ions were Cu_2_(IBU)_4_ and Cu_2_(IBU)_4_(CAF)_2_, respectively. The corresponding compounds with Ag(I) ions were Ag(IBU) and Ag(IBU)CAF. The structures of the obtained complexes are shown in Scheme 1.

**Figure 5:**
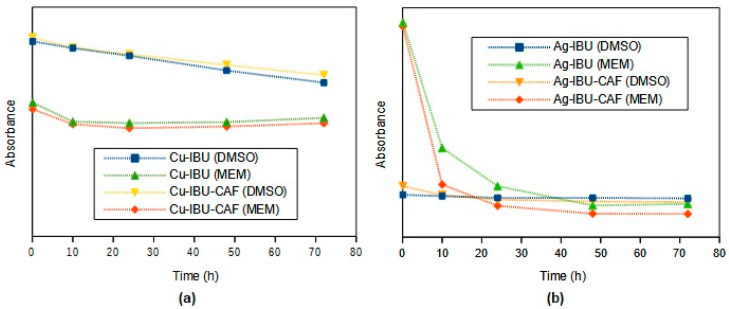
UV-VIS spectra in DMSO and Minimum Essential Medium Eagle (MEM) of: (**a**) complexes with Cu(II); and (**b**) complexes with Ag(I).

Since the complexes obtained were soluble in DMSO, and this solvent is often used in biological experiments, assessing the stability of these compounds in DMSO, as well as in its mixture with Minimum Essential Medium Eagle (MEM), was important. The spectroscopic measurements were performed at 266 nm, which is related to the π → π* transition band for IBU ligands [27,29]. It reflects the connection of IBU with the metal ion in the complexation. As shown in Figure 5, only in the case of the silver complexes in the MEM solution was a significant decrease in the absorbance value observed during the first 10 h of measurements. Thereafter, their band intensities changed very little and settled down after 48 h. When DMSO was used as a solvent, the absorbance value remained relatively constant for both silver complexes throughout the measurements.

For the copper complexes introduced into the MEM solution, the measured absorbance value decreased slightly during the first 10 h and remained constant thereafter, while a steady decrease in metal–IBU interactions was observed for the DMSO solution. 

This led to the conclusion that the obtained silver complexes were more stable in DMSO than in MEM. In contrast, all tested copper complexes decomposed slowly in DMSO, but their stability in MEM was higher.

### 2.2. In Silico ADME Prediction

Since the ADMET (absorption, distribution, metabolism, excretion, toxicity) prediction of drug candidates using in silico and in vitro methods has proved useful in the selection of newly prepared compounds, these tools were used for the preliminary evaluation of the obtained metal complexes. The in silico results given below were supplemented with in vitro cytotoxicity studies presented in the next parts of the work. The physiochemical properties of the studied compounds are given in Table 2.

The following basic pharmacokinetic parameters were estimated: aqueous solubility (ESOL—Estimated SOLubility); lipophilicity (MLOGP—Moriguchi octanol–water partition coefficient); absorption from the gastrointestinal tract (GI absorption); penetration of the blood–brain barrier (BBB permeability); and, finally, the evaluation of drug-likeness according to Lipinski’s rules (Table 3). The predicted parameters indicated that both Ag-based complexes could be considered as drug candidates.

### 2.3. Cytotoxicity Evaluation

To evaluate the safety of the newly obtained metal complexes for normal human cells, two different parameters were tested: cell proliferation with the use of the XTT test and the ability of viable cells to incorporate and bind the neutral red dye in the lysosomes with the NR test. The range of concentrations used here (17.5–140 μg mL^−1^) was based on similar studies of various complexes available in the literature [15] and our preliminary tests. Additionally, we assessed whether exposure to the studied complexes did not cause morphological changes in the cells.

**Figure 6:**
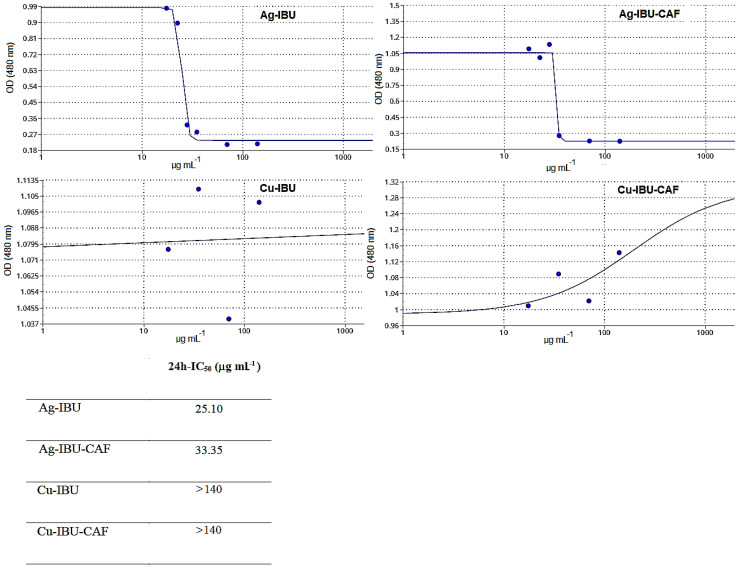
Dose–response curves based on the reaction of the CCD-18Co cells to the studied complexes and their estimated IC_50_ values after 24 h incubation derived from the XTT test (*n* = 3).

**Figure 7:**
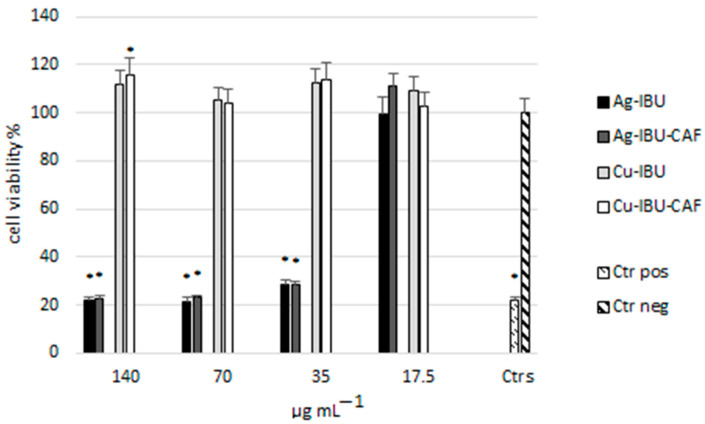
Effect of the tested complexes on the proliferation of CCD-18Co cells after 24 h exposure assessed by the XTT assay. Ctrs—controls, Ctr neg—negative control, Ctr pos—positive control (mean ± SD, * significantly different from negative control at *p* < 0.05).

**Figure 8:**
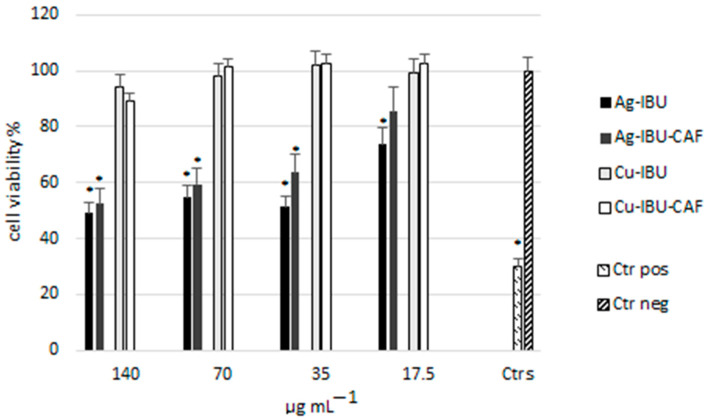
Effect of the tested complexes on the number of CCD-18Co cells after 24 h exposure assessed by the NR assay. Ctrs—controls, Ctr neg—negative control, Ctr pos—positive control (mean ± SD, * significantly different from negative control at *p* < 0.05).

**Figure 9:**
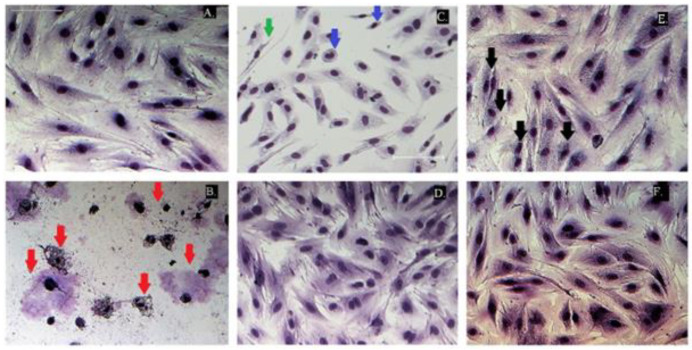
Effect of the tested complexes on morphology of CCD-18Co cells after 24 h exposure: (**A**) control; (**B**) Ag-IBU 70 μg mL^−1^; (**C**) Ag-IBU 17.5 μg mL^−1^; (**D**) Ag-IBU-CAF 17.5 μg mL^−1^; (**E**) Cu-IBU 70 μg mL^−1^; (**F**) Cu-IBU-CAF 70 μg mL^−1^. Arrows: red—necrotic/lysed cells, blue—apoptotic cells, green—elongated cells, black—intracellular granulation. Scale bar 100 μm.

The tested complexes showed varied effects on healthy fibroblast viability, with stronger cytotoxic effects at similar concentrations for Ag- compared to Cu-based complexes. The IC_50_ values for Ag-IBU and Ag-IBU-CAF were estimated at 25.10 and 33.35 μg mL^−1^, respectively (Figure 6). The lowest tested concentrations were found to be non-toxic or of low influence (Figure 7 and Figure 8). On the contrary, the Cu-based complexes were found to be less cytotoxic, with an IC_50_ above 140 μg mL^−1^. Only at the highest concentrations tested (70 and 140 μg mL^−1^) were some subtle changes in the microscopic images of cells exposed to Cu-containing complexes observed (Table 4; Figure 9).

At the higher tested concentrations, both types of Ag complexes (with IBU and with IBU and CAF) caused visible changes in the morphology, including cell elongation, intracytoplasmic granules, and evident signs of lysis and necrotic changes (Table 4, Figure 9). The total cell number also decreased (Figure 7). This indicated the moderate or severe cytotoxicity of the tested Ag complexes at high concentrations.

**Figure 10:**
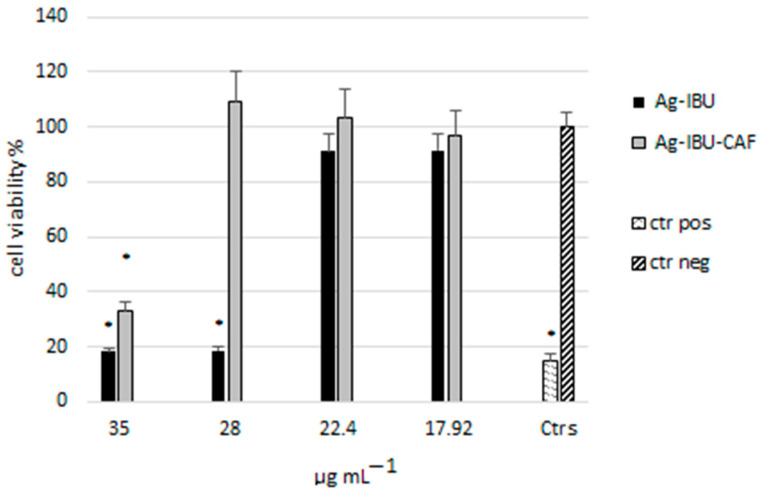
Effect of Ag-IBU and Ag-IBU-CAF at the narrowed range of concentrations on proliferation of CCD-18Co cells after 24 h exposure assessed by the XTT assay. Ctrs—controls, ctr neg—negative control, ctr pos—positive control (mean ± SD, * significantly different from negative control at *p* < 0.05).

Because of the high cytotoxicity of Ag-IBU and Ag-IBU-CAF, additional tests were performed with a narrow range of concentrations (17.92–35 μg mL^−1^), which showed evident differences in toxicity between these two silver-based complexes, with a stronger impact from the complex containing IBU only (Figure 10).

Such differences between the effects of complexes containing both IBU and CAF and those containing only IBU were visible in both Cu- and Ag-based compounds (Figure 9). In both cases, the presence of CAF weakened the cytotoxic effect on healthy fibroblasts.

Cu-based complexes showed lower cytotoxicity. Only at the highest concentrations tested (70 and 140 μg mL^−1^) were some subtle changes in the microscopic images of the exposed cells observed (Table 4). The differences between the morphologies of cells exposed to Cu-IBU or Cu-IBU-CAF at a concentration of 70 μg mL^−1^ are shown in Figure 9, where in image (E) there is visible intracellular granulation in some cells (black arrows). The increased appearance of such stress granules is a commonly observed response associated with cell defense against harmful stimuli. Cells exposed to the same concentration of Cu-IBU-CAF showed no differences compared to the control population (Figure 9A,F). 

The general observation was that complexes containing IBU and CAF had lower toxicity than those associated with IBU only. It can be assumed that these results are related to the cytoprotective effects of CAF, as similar effects were previously described after the exposure of fibroblasts to this alkaloid [33]. It was reported that in the cells exposed to various types of unfavorable stimuli, CAF was able to modulate ROS generation, protect cells against necrosis, or inhibit the excessive activation of fiber synthesis. 

The effect of the obtained complexes on the release of the pro-inflammatory cytokine TNF-α from the exposed cells, as a marker of pro-inflammatory activity, was evaluated. Studies were conducted with the use of the lowest concentration (17.5 µg mL^−1^), due to the high cytotoxicity of silver-based complexes at higher concentrations. None of the tested samples caused statistically significant changes in the TNF-α level compared to the negative control.

The obtained complexes were also examined for their anti-inflammatory activity as cyclooxygenase inhibitors. This enzyme, occurring in two isoforms, a constitutive COX-1 and an inducible COX-2 form, is responsible for the formation of prostaglandins from arachidonic acid. As prostaglandins are mediators of inflammation, inhibiting COX can alleviate its symptoms. Most NSAIDs, including IBU, act as non-selective COX inhibitors. Due to the involvement of COX-1 in many important cellular processes, a desirable feature of anti-inflammatory drug candidates is the ability to selectively inhibit COX-2, the action of which is closely correlated with the inflammatory process.

The effects of the tested complexes on COX activity are shown in Table 5.

Although both Cu complexes were not toxic to healthy fibroblasts, at the same time their anti-inflammatory activity was quite weak, with relatively high IC_50_ values toward both COX-1 and COX-2. There was no clear selectivity towards any form of COX. Similarly, IBU is classified as a non-specific COX inhibitor [34] or an inhibitor with a stronger effect on COX-1 [35]. However, in the case of the Ag-containing complexes, it was observed that the IBU- and CAF-containing combination had stronger COX-2 inhibitory activity, with an IC_50_ of 26.5 μg mL^−1^ compared to 62.5 μg mL^−1^ for COX-1, and an SI value of 2.4. This value still does not indicate high selectivity toward COX-2, but such a shift in the activity of this complex is very interesting and desirable. This may be explained by the presence of CAF, as its adjuvant analgesic effect in combination with various analgesic drugs was described previously [9]. CAF alone does not have a strong COX-2 inhibitory effect, but it has been observed that combining it with, e.g., acetylsalicylic acid produces a synergistic effect in inhibiting the synthesis of PGE2 [9,36]. CAF is a non-selective antagonist of adenosine receptors, including the A2α receptors involved in the upregulation of the COX-2 gene, which may explain the observed effects [37,38]. 

Additionally, while a relatively strong cytotoxic effect of the Ag complexes was observed in the tested concentrations, and, compared to the toxic concentrations, the IC_50_ for COX-2 was still relatively high, the COX-2 IC20 had a much lower value, amounting to 13.3 μg mL^−1^ for Ag-IBU and 1.6 μg mL^−1^ for Ag-IBU- CAF. 

Several metal complexes based on more than twenty different metals, exerting functions which cannot be performed by any organic molecule, have already been approved for medicinal uses [39]. As was previously confirmed, both IBU and CAF can form complexes with different substances to improve their physicochemical properties [2]. Thus, the goal of this research was to obtain new combinations with selected metals, i.e., Ag and Cu. The properties of the Ag-IBU-CAF complex in terms of its activity and biosafety were particularly interesting because, to our knowledge, similar studies have not been conducted in this direction so far. 

The solubility of a drug is one of the most important factors determining the speed and amount of its absorption, along with its bioavailability [40]; therefore, attempts are being made to improve this parameter, for example, by creating various types of drug combinations, including caffeine complexation. There are a number of methods to increase the solubility of medicinal substances, but they may entail a change in the safety profile of the drug. This requires a thorough assessment of the cytotoxicity of the final product to be used each time. 

Both Ag and Cu can be toxic, while in the form of complexes with various ligands this toxicity can be changed, so different combinations can show many beneficial and desirable properties, enabling them to be used in therapy [41]. Ag, belonging to the group of non-essential metals, is generally more toxic and can cause detrimental effects at lower concentrations than the essential metals, including Cu; however, the latter can also cause adverse effects when threshold concentrations are exceeded [42]. The presented studies also showed a difference in the cytotoxicity of the complexes based on these two metals. For this reason, it is important to estimate safe concentrations and assess whether the tested drugs/drug complexes will meet the assumed therapeutic effects at a given concentration. This study was also undertaken because of the relatively few studies on the cytotoxicity of Ag and Cu complexes with IBU against normal human cells, as most in vitro studies have been performed on transformed cells.

Ag complexes with different ligands have gained attention due to the possibility of using them in the treatment of various diseases, in particular cancer [43]. The activity of Ag, thanks to which its various forms are used as antibacterial, fungicidal, and anticancer agents, is based to a large extent on oxidative shifting in the redox status of exposed cells and is modulated by the presence of the specific ligands, regulating lipophilicity, water solubility, stability, and the rate of ion release [44,45,46].

The cytotoxicity of Ag complexes with various ligands, including several NSAIDs, against cancer cells has been observed in many studies [13,43,45,46]. It was reported that the mechanisms of the influence of Ag-NSAID combinations on these cells involve the induction of ROS generation, the overloading of calcium, activating caspase-3, and mitochondrial damage, with the concomitant inhibition of various antioxidant defense enzymes, leading to apoptosis [44,47]. Moreover, some studies have proved that such cytotoxic activity is selectively directed against cancer cell lines, or at least that the IC_50_ values for cancer cells and healthy cells differ significantly, which may allow the use of such complexes in therapy without strong side effects [43,45,47]. However, this seems to depend mostly on the type of complex and, to some extent, on the cells used, as some IC_50_ values reported for normal cells were also relatively low [13]. Generally, there are not many studies on the safety of such complexes based on healthy cells.

In any case, the biosafety assessment requirements for any newly developed product with potential therapeutic use are regulated by the International Organization for Standardization guidance, with Part 5 containing guidance on in vitro cytotoxicity assessment (ISO-10993-5:2009) [48]. This standard indicates the XTT and NR tests as suitable means for quantitative cytotoxicity determination. Additionally, qualitative determination by cell morphology examination is suggested. 

The conducted research showed that the complexes with Ag(I) had a stronger suppressive effect than the analogous complexes with Cu(II). Another observation was that the addition of CAF to the complex reduced the cytotoxicity of the tested substances compared to complexes containing only IBU, which seemed to result from the protective effect of CAF on exposed cells. This effect appears to be very interesting and worthy of further research.

The toxicity of Cu-based complexes is connected with ligands, required for therapeutic effects [49]. However, similarly, data on the cytotoxic effects of Cu ions and various Cu complexes largely refer to studies using cancer cell lines rather than healthy ones. An IC_50_ value as low as 15 μg mL^−1^ of Cu(II) ions was calculated for the human embryo cell lines CLV102 and Lu106 [50]. It is also known that free Cu ions can be cytotoxic due to their high redox activity, and thus the intracellular concentration of this metal has to be kept at the appropriate level [51]. For this reason, research on the safety of Cu-based complexes in relation to healthy cells in the case of the synthesis of new forms should be comprehensively carried out. No similar studies have been found in the literature on Cu or Ag complexes with IBU or CAF in terms of their cytotoxic effect on healthy cells.

### 2.4. Antibacterial Effect

Ten bacterial strains divided into Gram-positive (*E. faecalis* ATCC 29212, *C. bifermentans* ATCC 638, *B. subtilis* ATCC 6633, *S. aureus* ATCC 6538, *S. aureus* ATCC 25923, *S. aureus* ATCC 29213, *S. aureus* ATCC 43300, and *S. epidermidis* ATCC 12228) and Gram-negative (*B. fragilis* ATCC 25285, *E. coli* ATCC 35218, and *P. aeruginosa* ATCC 27853) were included in this study to analyze the ability of all four complexes to affect the growth of these microorganisms.

The antibacterial activity of the metal complexes was first evaluated by the microdilution method on 96-well plates to determine the MIC values. To this end, all bacteria were incubated with the complexes in a concentration range between 2.5 and 160 µg mL^−1^ (2.5, 5, 10, 20, 40, 80, 160). The MIC values for the Ag-IBU and Ag-IBU-CAF complexes were determined at concentrations of 20 µg mL^−1^ or lower in the case of all tested bacterial strains. The effects of the metal complexes against different bacterial strains are listed in Table 6. The results for Ag-IBU were slightly weaker when compared with Ag-IBU-CAF. The MIC values were lower in the case of Gram-positive bacteria, but this phenomenon would probably not be the rule if other bacteria were included in this analysis. Unfortunately, no inhibitory effect was measured for Cu-IBU and Cu-IBU-CAF up to a complex concentration of 160 µg mL^−1^. The antibacterial effects were compared with those of IBU. Even at concentrations of more than 200 µg mL^−1^, no inhibitory effect was determined. This is consistent with other reports [27]. The Ag nanoparticles functionalized with PSBA (Ag NPs@PSBA) described by Amin et al. [26], when tested using clinically isolated strains of both *E. coli* and *S. aureus*, possessed similar antibacterial activity according to the estimated MICs. In comparison, in our study, IBU alone did not show antimicrobial activity, whereas in complex with Ag and CAF it showed an increasing influence on microbial growth. In the case of Ag NPs@PSBA, the antibacterial effect increased when compared to PSBA alone according to the estimated zones of inhibition.

The next step was the determination of the inhibition halo using the Kirby–Bauer method. Ag-IBU and Ag-IBU-CAF were tested with each of the ten bacteria. The complex concentrations were chosen based on the estimated MIC. In almost all cases, the inhibition zone was at least 10 mm, and in a few cases even 15–16 mm, meaning that the complexes were quite active at these concentrations.

The determined MIC values were lower than the IC_50_ values estimated in the proliferation assays with CCD-18Co cells (Figure 10).

## 3. Materials and Methods

### 3.1. Synthesis Procedure

All reagents and solvents used in this study were purchased from Merck (Sigma-Aldrich, St. Louis, MO, USA) and then used without further purification. 

#### 3.1.1. Cu-IBU

The sample was prepared according to the procedure of Trinchero et al. [18] using CuSO_4_·5H_2_O as a copper source. The copper sulphate pentahydrate (0.250 g) and ibuprofen sodium salt (IBU-Na) (0.456 g) were separately dissolved in distilled water, mixed, and stirred for 30 min. The suspension was kept at 4 °C overnight. Next, the precipitate was filtered and washed several times with distilled water. Elemental analysis: Calculated (%)—C, 65.87; H, 7.24. Found (%)—C, 65.78; H, 7.64. Molar conductivity Λ_m_ (0.001 M in DMSO)—3.1 S cm^2^ mol^−1^.

#### 3.1.2. Cu-IBU-CAF

The complex was synthesized with CuSO_4_·5H_2_O (0.250 g), ibuprofen sodium salt (IBU-Na) (0.456 g), and CAF (0.194 g). Each compound was dissolved in an aqueous methanol solution (with a volume ratio of 1:1). The copper salt solution was heated to 50 °C, after which the ibuprofen and caffeine solutions were dropped into it. The resulting mixture was stirred for 30 min and then left at 4 °C overnight. After that, the precipitate was filtered and washed several times with distilled water. Elemental analysis: Calculated (%)—C, 61.10; H, 6.65; N, 8.38. Found (%)—C, 60.75; H, 7.19; N, 8.41. Molar conductivity Λ_m_ (0.001 M in DMSO)—2.2 S cm^2^ mol^−1^.

#### 3.1.3. Ag-IBU

The sample was prepared according to the slightly modified procedure of Pereira e Silva [27]. The salt solutions were heated to 50 °C and mixed. After stirring for 30 min, the mixture was left at 4 °C overnight. Next, it was filtered and washed with distilled water. Elemental analysis: Calculated (%)—C, 49.86; H, 5.48. Found (%)—C, 50.13; H, 5.78. Molar conductivity Λ_m_ (0.001 M in DMSO)—3.6 S cm^2^ mol^−1^.

#### 3.1.4. Ag-IBU-CAF

The compound was synthesized with AgNO_3_ (0.170 g), ibuprofen sodium salt (IBU-Na) (0.228 g), and CAF (0.194 g) and then dissolved in an aqueous methanol solution (with a volume ratio of 1:1). The silver salt solution was heated to 50 °C, after which the ibuprofen and caffeine solutions were dropped into it. The resulting mixture was stirred for 30 min and then left at 4 °C overnight. After that, the precipitate was filtered and washed several times with distilled water. Elemental analysis: Calculated (%)—C, 49.71; H, 5.38; N, 11.04. Found (%)—C, 50.09; H, 5.67; N, 10.72. Molar conductivity Λ_m_ (0.001 M in DMSO)—2.4 S cm^2^ mol^−1^.

### 3.2. Material Characterization

Elemental analysis was conducted using a Perkin Elmer 2400 Analyser (Perkin Elmer, Waltham, MA, USA).

Conductivity measurements were carried out at room temperature using a CPV-411 conductivity meter (Elmetron, Zabrze, Poland) with an ECF-1 electrode. Molar conductivities were obtained for freshly prepared 0.001 M DMSO solutions. Conductometric titration was performed between an aqueous IBU-Na solution of 0.005 M and an aqueous salt solution (AgNO_3_ or CuSO_4_) of 0.01 M. Conductivity was recorded after every addition of 1 mL of metal salt solution to 25 mL of ligand solution. The results obtained were corrected for dilution.

The FTIR spectra were recorded at room temperature on a Nicolet 6700 spectrometer (Thermo Scientific™, Waltham, MA USA) using the smart iTR diamond ATR method in the range of 600–3500 cm^−1^ at a 4 cm^−1^ resolution. 

TG analyses for all samples were performed in ceramic crucibles using a Thermogravimeter Q-1500D instrument (MOM, Budapest, Hungary) in the temperature range of 25–700 °C under an air flow, at a heating rate of 10 °C min^−1^.

X-ray measurements were performed using an Empyrean (PANalytical, Malvern, UK) diffractometer in a wide range of diffraction angles (from 3.5° to 70°, with a step size of 0.013°). The X-ray analysis system was equipped with a line focus Cu X-ray tube, Ni filter, generator settings of 40 kV and 40 mA, and a PIXcel3D detector for the scanning line 1D detector mode.

The stability studies were conducted in DMSO and cell growth medium solutions. The stock solutions of each complex were made by dissolving 5 mg of the sample in 1 mL DMSO. Next, 0.1 mg mL^−1^ solutions in DMSO or Minimum Essential Medium Eagle (MEM) were prepared. UV-VIS measurements were performed after 5 min, 10 h, 24 h, 48 h, and 72 h using a UV-1800 spectrometer (Shimadzu, Kyoto, Japan) at a 266 nm wavelength.

### 3.3. In Silico ADME (Absorption, Distribution, Metabolism, Excretion) Prediction

The prediction of the ADME profiles of the obtained complexes was conducted using the web application SwissADME (http://www.swissadme.ch/, accessed on 18 December 2023/).

### 3.4. Cytotoxicity

#### 3.4.1. Cells and Culture Conditions

The cell line CCD-18Co (fibroblasts from the colon of a healthy human) was obtained from the American Type Cells Collections (Cat. No. ATCC-CRL-1459). The cells were cultured in Minimum Essential Medium Eagle (MEM) with Earle’s salts, L-glutamine, sodium bicarbonate, and phenol red, supplemented with 10% fetal bovine serum (FBS), 1% non-essential amino acids (NEAAs), and 1% penicillin/streptomycin solution (P/S), all purchased from Sigma-Aldrich (St. Louis, MO, USA). The incubation parameters were 37 ℃, 5% CO2, and 95% relative humidity.

#### 3.4.2. Cytotoxicity Assays

The cytotoxicity of the tested complexes (Ag-IBU, Ag-IBU-CAF, Cu-IBU, and Cu-IBU-CAF) was derived using two distinct parameters: (1) tetrazolium salt (XTT) reduction by mitochondrial succinate dehydrogenases using an XTT (Tetrazolium hydroxide) Kit; and (2) neutral red (NR) dye uptake and incorporation within lysosomes using an NR (Neutral Red) Kit. Both kits were produced by Xenometrix AG, Allschwil, Switzerland. The assays were carried out without any substantial changes to the test procedures provided by the manufacturer. 

The stock solutions of the tested compounds at 20 mg mL^−1^ were prepared in DMSO. The working suspensions were subsequently prepared in MEM. Cells were seeded at 1 × 105 per 1 mL in 96-well plates at a volume of 200 µL and left overnight to adhere. Thereafter, the medium was removed, and the cells were treated with Ag-IBU, Ag-IBU-CAF, Cu-IBU, and Cu-IBU-CAF at concentrations of 0–140 µg mL^−1^ for 24 h. Due to the higher cytotoxicity of the Ag-IBU and Ag-IBU-CAF complexes, additional determinations were also made using a narrowed range of concentrations. Fresh medium alone was used in the negative control samples and with the addition of 0.5% Triton X-100 (Sigma-Aldrich, St. Louis, MO, USA) for the positive control.

For the XTT assay, the exposure medium was collected for subsequent TNF-α determination, and fresh MEM containing a mixture of XTTII and XTTI reagents (2,3-bis [2-methoxy-4-nitro-5-sulfopheny]-2H-tetrazolium-5-carboxyanilide inner salt and buffer) in a 1:100 ratio was added. The cells were incubated for the next 3 h, gently mixed, and the absorbance was read at 480 nm with a reference wavelength of 690 nm using the multi-mode plate reader FLUOstar Omega (BMG Labtech, Ortenberg, Germany).

For the NR assay, the exposure medium was removed, and each well was washed with the wash solution. Then, the cells were labeled with the labeling solution (NR, diluted 1:200 in the culture medium) for 3 h. Unbound dye was washed off with PBS, and cells were fixed. After solubilization, the absorbance was read at 540 nm with a reference wavelength of 690 nm using the multi-mode plate reader.

All solutions used for the XTT and NR assays were included in the kits. Experiments were conducted with at least two independent repetitions of the whole procedure. Each determination was performed with three replicates. The 50% inhibitory concentration (IC_50_) was calculated from the concentration–response curves (Quest Graph™ IC_50_ Calculator; AAT Bioquest, Inc.; https://www.aatbio.com/tools/ic50-calculator, accessed on 2 July 2023).

Additionally, the morphology of cells exposed to the studied complexes for 24 h was assessed. For this purpose, after the exposure, the medium was removed, and the cells were fixed and stained using a Hemacolor staining kit (Sigma-Aldrich, St. Louis, MO, USA) containing eosin and azure solutions. After drying, the cells were analyzed with a Nikon Eclipse Ti inverted microscope. Any changes in cell morphology were assessed and graded according to the scores given in Table 7.

The statistical analysis of the data was performed using Statistica 10.0 software (StatSoft Inc., Tulsa, OK, USA). One-way analysis of variance followed by Tukey’s HSD test was used. *p* < 0.05 was considered to indicate statistical significance.

#### 3.4.3. Pro- and Anti-Inflammatory Assays

TNF-α levels in supernatants collected after the exposure of the cells to lower concentrations of the studied complexes (17.5 µg mL^−1^, 24 h of the exposure) were tested with a Human TNF-α ELISA kit (Invitrogen, Waltham, MA USA) according to the manufacturer’s instructions. Cytokine levels in the studied samples were calculated using the equation obtained from the standard curve prepared using standard solutions of TNF-α.

The ability of the obtained complexes to inhibit cyclooxygenase activity was measured using a COX (ovine/human) Inhibitor Screening Assay Kit (Cayman Chemicals, MI, USA) with ovine COX-1 and human recombinant COX-2. The amount of prostaglandin, dependent on the COX activity, after exposure to the tested complexes was calculated from the standard curve. The concentrations of the compounds causing 50% and 20% inhibition (IC_50_, IC_20_) were calculated from the concentration–inhibition response curve (Quest Graph™ IC_50_ Calculator; AAT Bioquest, Inc.; https://www.aatbio.com/tools/ic50-calculator, accessed on 7 December 2023). The selectivity indices (SIs) were calculated according to the formula COX-1 IC_50_/COX-2 IC_50_.

### 3.5. Antibacterial Assays

#### 3.5.1. Bacterial Strains

The bacterial strains *Bacillus subtilis* ATCC 6637, *Bacteroides fragilis* ATCC 25285, *Clostridium bifermentans* ATCC 638, *Escherichia coli* ATCC 35218, *Enterococcus faecalis* ATCC 29212, *Pseudomonas aeruginosa* ATCC 27853, *Staphylococcus aureus* ATCC 6538, *Staphylococcus aureus* ATCC 25923, *Staphylococcus aureus* ATCC 29213, *Staphylococcus aureus* ATCC 43300, and *Staphylococcus epidermidis* ATCC 12228 were cultured in Mueller–Hinton medium (Oxoid, Basingstoke, UK) at 37 °C for 24 h.

#### 3.5.2. Minimal Inhibitory Concentration

The minimal inhibitory concentration (MIC) was determined by the broth microdilution method according to the standards of the Clinical and Laboratory Standards Institute (CLSI) manuals in 96-well plates. All stock solutions of the investigated complexes were prepared in DMSO with concentrations of 2 mg mL^−1^.

The MIC was estimated as the lowest concentration of the complex inhibiting bacterial growth. As negative controls, samples without inoculum at each complex concentration were used, whereas the positive control contained the inoculum but not the investigated complexes. Each complex was tested at concentrations between 2.5 and 160 µg mL^−1^. The first well contained the highest concentration, and further concentrations were achieved by serial dilution. The assay was performed in duplicate with three repetitions for each bacterial strain.

#### 3.5.3. Disc Diffusion Test

The antimicrobial sensibility assay was performed according to the Kirby–Bauer method using 6 mm filter discs. Mueller–Hinton agar (Oxoid) was poured onto Petri dishes (90 mm), and saturated filter discs at the concentration determined as the MIC were placed onto the agar surface after distributing bacteria on the agar. The Petri dishes were incubated at 37 °C for 24 h.

## 4. Conclusions

Newly synthesized binary complexes of metal ions—Cu(II) and Ag(I)—with IBU and ternary complexes with IBU and CAF were subjected to physicochemical and biological analysis. The copper complexes showed higher stability in MEM solution, while the silver complexes were more stable in DMSO. The Cu-based complexes were less toxic to healthy human fibroblasts; however, their anti-inflammatory activity was also weaker compared to the Ag-based complexes. Furthermore, complexes containing IBU and CAF exhibited lower toxicity than those associated with IBU only. Additionally, the Ag-IBU and Ag-IBU-CAF complexes were found to exhibit good antibacterial properties against several strains. The determined MIC values were below the concentrations estimated in the in vivo toxicity tests. Taking into account the interesting features of the obtained complexes, mainly Ag-IBU-CAF, it seems that further research could be carried out regarding their potential application in preparations used, for example, in the healing of superinfected wounds.

## Figures and Tables

**Table 1:** Wavenumbers and relative Δν values obtained using FTIR method.

Sample	Wavenumber (cm^−1^)		
	*ν*_asym_	*ν*_sym_	Δ*ν*
Na-IBU	1581	1400	181
Ag-IBU	1531	1380	151
Ag-IBU-CAF	1530	1380	150
Cu-IBU	1584	1404	180
Cu-IBU-CAF	1584	1404	180

**Table 2:** Physicochemical properties of obtained metal complexes.

Sample	Molecular Weight (g mol^−1^)	Heavy Atoms	Fraction Csp3	Rotatable Bonds	H-Bond Donors	H-Bond Acceptors	Molar Refractivity	TPSA (Å^2^)
Cu-IBU	952.22	62	0.54	16	0	8	241.22	73.84
Cu-IBU-CAF	1346.64	90	0.59	18	0	16	368.50	168.04
Ag-IBU	314.15	16	0.54	4	0	2	60.47	18.46
Ag-IBU-CAF	508.34	30	0.48	4	0	5	112.50	80.28

**Table 3:** Predicted physicochemical descriptors of obtained metal complexes.

Sample	Solubility Log S (ESOL)	MLOGP	GI Absorption	BBB Permeant	Drug-Likeness (Lipinski)
Cu-IBU	−14.90	5.49	Low	No	No
Cu-IBU-CAF	−16.43	2.69	Low	No	No
Ag-IBU	−4.28	2.42	High	Yes	Yes
Ag-IBU-CAF	−5.31	2.21	High	No	Yes

**Table 4:** Grading of cytotoxicity of the studied complexes on CCD-18Co cells after 24 h exposure.

Concentration (μg mL^−1^)	Ag-IBU	Ag-IBU-CAF	Cu-IBU	Cu-IBU-CAF
140	4	4	1	1
70	4	3	1	1
35	3	2	0	0
17.5	2	1	0	0

**Table 5:** Effects of the metal complexes on COX isoform activity.

Sample	IC_50_ (μg mL^−1^)		SI *
	COX-1	COX-2	
Ag-IBU	64.6	65.5	1
Ag-IBU-CAF	62.5	26.5	2.4
Cu-IBU	72.6	78.8	0.9
Cu-IBU-CAF	78.8	65.2	1.2

* SI—COX-2 Selectivity Index (COX-1 IC_50_/COX-2 IC_50_).

**Table 6:** Effects of the metal complexes Ag-IBU and Ag-IBU-CAF on bacterial growth.

Bacterial Strain	MIC (µg mL^−1^)		Inhibition Halo (mm) *	
	Ag-IBU	Ag-IBU-CAF	Ag-IBU	Ag-IBU-CAF
*Pseudomonas aeruginosa* ATCC 27853	10	10	10	15
*Bacteroides fragilis* ATCC 25285	20	20	7	10
*Escherichia coli* ATCC 25218	10	5	9	17
*Clostridium bifermentans* ATCC 638	20	20	8	10
*Bacillus subtilis* ATCC 6637	10	10	11	13
*Enterococcus faecalis* ATCC 29212	10	5	14	16
*Staphylococcus aureus* ATCC 6538	10	5	14	15
*Staphylococcus aureus* ATCC 25923	10	5	14	16
*Staphylococcus aureus* ATCC 29213	5	5	7	12
*Staphylococcus aureus* ATCC 43300	20	10	13	15
*Staphylococcus epidermidis* ATCC 12228	20	10	10	11

* Concentration according to MIC.

**Table 7:** Qualitative morphological grading of cytotoxicity (adopted from ISO-10993-5:2009).

Grade	Effect	Cell Morphology
0	None	No substantial changes in cell morphology or number
1	Slight	Up to 20% round or detached cells, and/or slight changes in the morphology of a small part of the cells, and/or slight growth inhibition
2	Mild	Up to 50% round or detached cells, and/or some changes in the morphology of part of the cells, and/or less than 50% growth inhibition, no extensive cell lysis
3	Moderate	Up to 70% of cells rounded or lysed, more than 50% growth inhibition, but cells not completely destroyed
4	Severe	Nearly complete or complete destruction of the cells

## Data Availability

The data presented in this study are available from the authors on request.
